# Downregulation of GDF15 suppresses ferroptosis and predicts unfavorable prognosis in clear cell renal cell carcinoma

**DOI:** 10.1186/s13008-023-00103-9

**Published:** 2023-12-11

**Authors:** Dongliang Yang, Zhongyin He, Jiawei Lu, Xiaolin Yuan, Haiyong Liu, Yagang Xue, Ting Chen, Hongxing Gu

**Affiliations:** 1 Department of Urinary Surgery, The Affiliated Zhangjiagang Hospital of Soochow University, Suzhou, China; 2 Department of Oncology, The Affiliated Zhangjiagang Hospital of Soochow University, Suzhou, China

**Keywords:** ccRCC, GDF15, Ferroptosis, TCGA-KIRC, Biomarker

## Abstract

**Background:**

Growth differentiation factor 15 (GDF15), a member of the transforming growth factor beta (TGF-β) superfamily, is involved in various pathophysiological processes such as anorexia, obesity, inflammation, and tumorigenesis. However, the role of GDF15 in clear cell renal cell carcinoma (ccRCC) remains poorly understood.

**Methods:**

Clinical significance of GDF15 in ccRCC as well as other types of human cancers was analyzed using the TCGA PANCAN dataset. Gene Set Enrichment Analysis (GSEA) was used to study the significantly enriched pathways associated with GDF15 expression. qRT-PCR was used to quantitatively assess relative mRNA expression level. Flow cytometry was used to detect cell cycle. CCK-8 assay, colony formation assay, wound healing assay, Transwell migration/invasion assay, and EdU assay were used to comprehensively examine tumor viability and aggressiveness. MDA and iron assays were used to determine ferroptosis-related intracellular changes.

**Results:**

We found that GDF15 expression is decreased in renal carcinoma tissue. In 769-p and Caki-1 cells, GDF15 knockdown significantly promoted tumor viability, proliferation, and migration. Conversely, overexpression of GDF15 suppressed cell proliferation and invasion. Results from GSEA suggested that GDF15 might play a crucial role in ferroptosis. We further demonstrated that GDF15 is correlated with intracellular iron and lipid peroxidation MDA in 769-p and Caki-1 cells. In summary, we conclude that GDF15 inhibits migration and invasion of ccRCC cells by regulating ferroptosis.

**Conclusion:**

Our study demonstrates that GDF15 downexpression promotes viability and aggressiveness of ccRCC cells by abolishing ferroptosis, which confers unfavorable patient survival outcomes.

**Supplementary Information:**

The online version contains supplementary material available at 10.1186/s13008-023-00103-9.

## Introduction

Renal cell carcinoma (RCC) is a common malignant tumor of the urinary system and its incidence has been on the rise recently, affecting more than 1.2 million patients worldwide in the past 5 years [1, 2]. Clear cell renal cell carcinoma (ccRCC) is the most common subtype of RCC, accounting for approximately 80% to 90% of cases, and is the pathological type of the vast majority of metastatic RCC (mRCC) [3, 4]. The application of surgery and targeted therapy has improved the survival of ccRCC patients to some extent, but mortality remains high. Distant metastasis and recurrence are the main reasons for treatment failure in ccRCC patients. Studies have shown that renal cancer is a malignant tumor with strong proliferative and invasive potential, with 17% of patients having distant metastases at the time of initial diagnosis [3, 4]. Recent studies have demonstrated the effectiveness of immune checkpoint inhibitors in the treatment of ccRCC, nevertheless only a small subset of patients may benefit from immunotherapy [5]. Therefore, it is critical to further investigate the mechanisms of progression in ccRCC in order to develop more effective anti-tumor therapies.

Growth differentiation factor-15 (GDF15) is a cysteine knot protein of the transforming growth factor beta (TGF-β) superfamily [6]. The upregulation of GDF15 can be induced by cellular stress such as hypoxia, inflammation, and trauma, and the role of GDF15 in energy homeostasis has attracted significant attention in its potential for the treatment of cardiovascular diseases, metabolic disorders, and aging-related diseases [7–9]. Recent studies have demonstrated that GDF15 may also be implicated in a variety of neoplastic biological processes, including carcinogenesis and progression of non-small-cell lung carcinoma [10], cervical cancer [11], prostate cancer [12], hepatocellular carcinoma [13–15], colorectal cancer [16], and leukemia [17]. However, current studies on the role of GDF15 in cancer progression yielded conflicting results and, to date, the role of GDF15 in ccRCC and the underlying mechanisms are lacking.

Here, we discovered that GDF15 downregulation, an event highly specific to ccRCC, was associated with worse prognosis. GDF15 downregulation contributed to tumor viability and aggressiveness and was shown to significantly hinder ferroptosis in ccRCC. These results offer a novel insight into the important role of GDF15 in regulating ferroptosis during the initiation and progression of ccRCC.

## Results

### GDF15 is downregulated in ccRCC in a tissue-dependent and cancer type-specific fashion

**Fig. 1:**
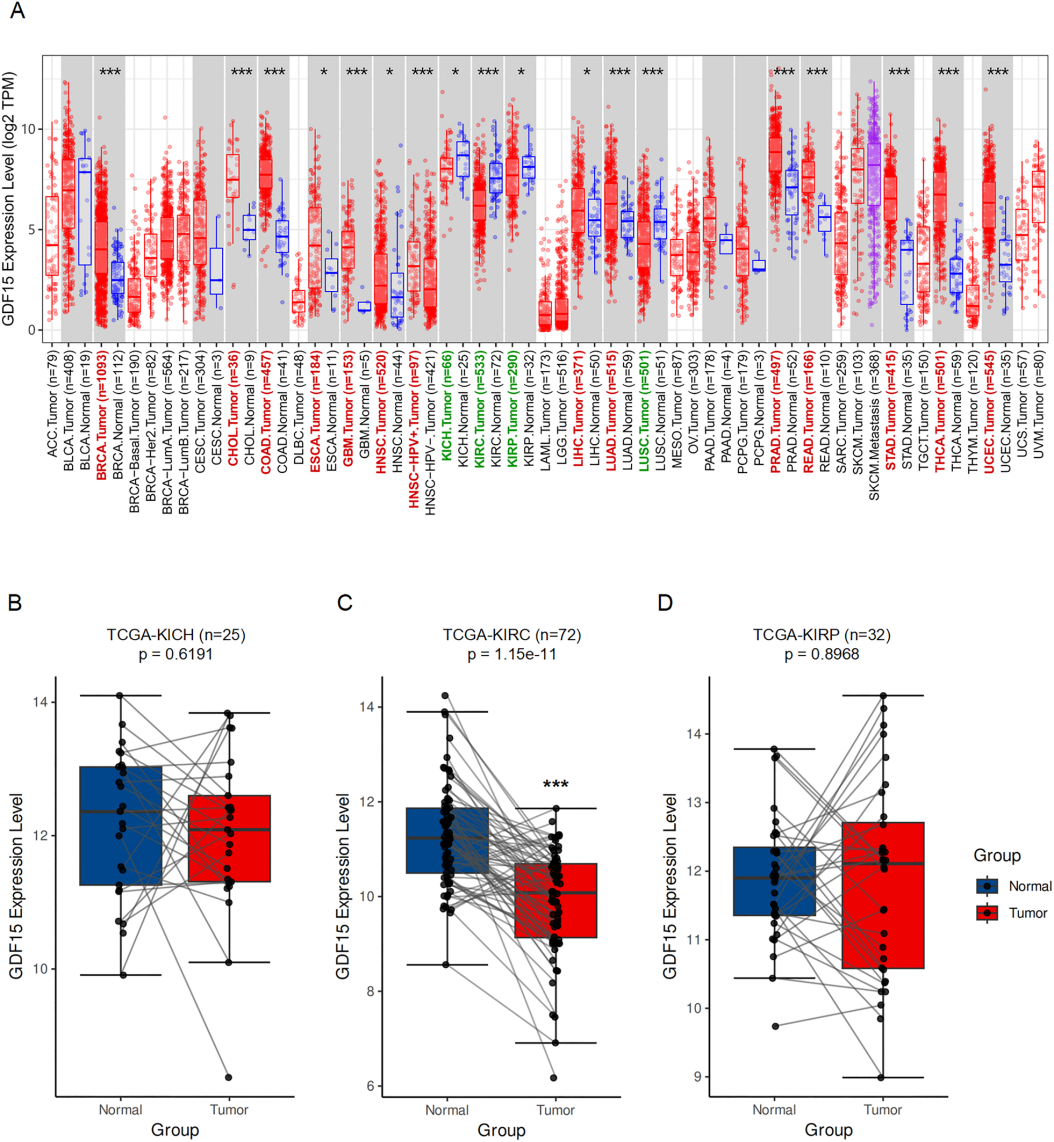
Investigation of differential GDF15 expression across different tumors. **A.** Box plots showing the differential expression between tumor and adjacent normal tissues for GDF15 across all TCGA tumors. Tumor types with normal data available are displayed in gray columns. The statistical significance is computed by Wilcoxon test. **B**-**D**. Box plots showing differential expression of GDF15 in chromophobe renal cell carcinoma (KICH, **B**), clear-cell renal cell carcinoma (KIRC, **C**), and papillary renal cell carcinoma (KIRP, **D**) tissue samples compared to their corresponding normal counterparts. The statistical significance is computed by paired t-test, with Welch-Satterthwaite approximation used to estimate the ‘effective’ degrees of freedom. * p < 0.05, *** p < 0.001

In order to systematically analyze the role of GDF15 in cancer, we utilized TCGA PANCAN dataset and looked at the expression level of GDF15 mRNA across different types of tumors. We found that the expression level of GDF15 in tumor samples, compared to that in normal samples, was significantly decreased in kidney malignancies, including chromophobe renal cell carcinoma (KICH), clear-cell renal cell carcinoma (KIRC), and papillary renal cell carcinoma (KIRP) (Fig. 1A). In contrast, GDF15 was frequently upregulated in other tumors, including breast invasive carcinoma (BRCA), cholangiocarcinoma (CHOL), colon carcinoma (COAD), esophageal carcinoma (ESCA), glioblastoma multiforme (GBM), head and neck squamous cell carcinoma (HNSC), liver hepatocellular carcinoma (LIHC), lung adenocarcinoma (LUAD), prostate adenocarcinoma (PRAD), rectum adenocarcinoma (READ), stomach adenocarcinoma (STAD), thyroid carcinoma (THCA), and uterine corpus endometrial carcinoma (UCEC) (Fig. 1A). These results suggested that GDF15 may be specifically downregulated in kidney tumors, whereas in other types of tumors it is usually upregulated.

Next, we analyzed the three kidney tumor datasets in TCGA dataset by selecting only the patients with RNAseq data available for both their tumor samples and the corresponding adjacent normal tissue samples (Fig. 1B-D), while filtering out those with RNAseq data available for their tumor samples only. Utilizing paired t’-test, we discovered that GDF15 expression level was not statistically different between the tumor and paired normal samples in TCGA-KICH (Fig. 1B) and TCGA-KIRP (Fig. 1D). Nevertheless, GDF15 was significantly downregulated in ccRCC samples compared to their adjacent normal tissue samples (Fig. 1C). Taken together, these results indicated that decreased expression of GDF15 is a hallmark highly specific to ccRCC and should be prioritized for prognostic and mechanistic research.

### GDF15 downregulation in ccRCC is validated in clinical specimens and associated with unfavorable prognosis

**Fig. 2:**
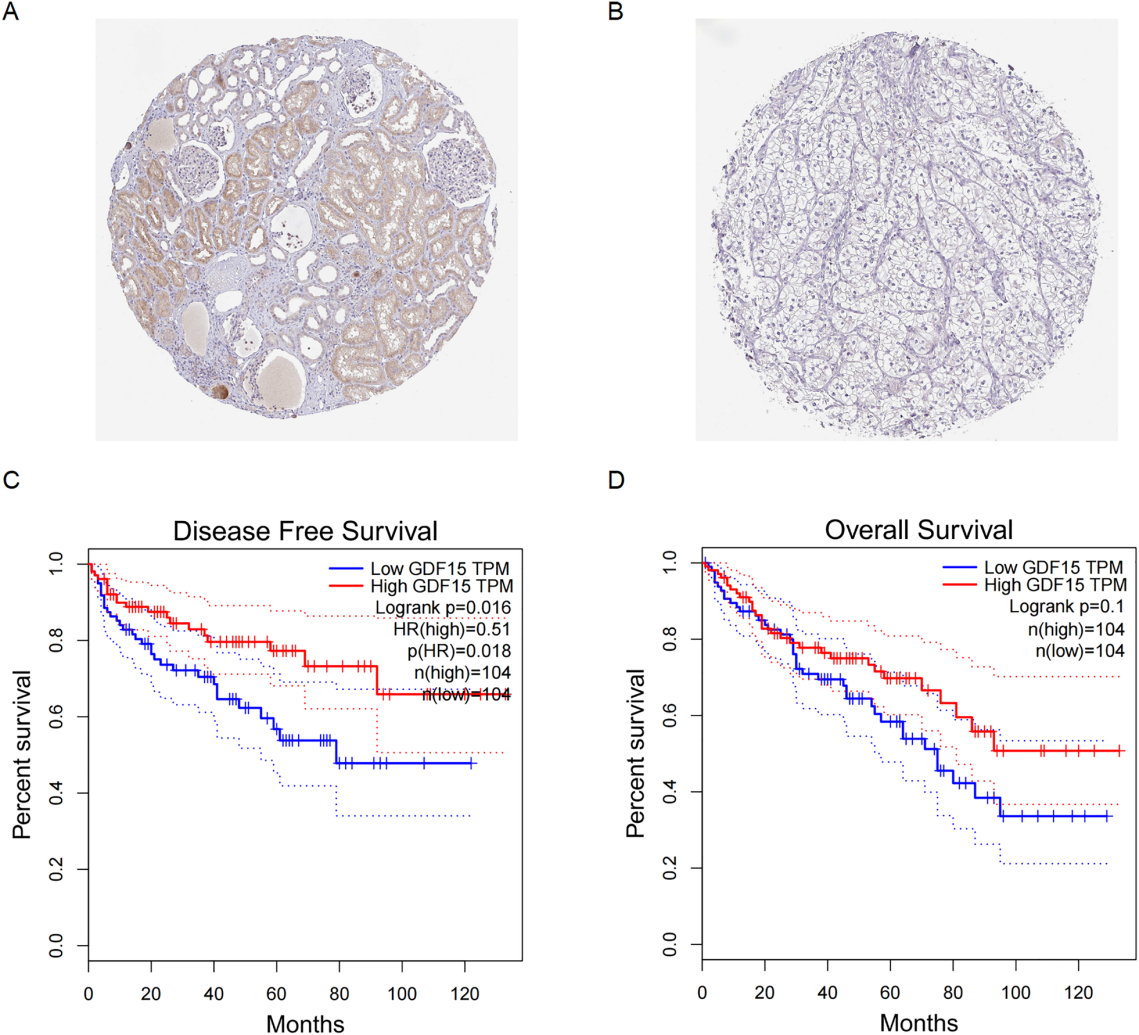
Validation of GDF15 downregulation in ccRCC tumor specimens and its clinical significance. **A.** Representative immunohistochemistry figure of normal kidney sample in the Human Protein Atlas. **B.** Representative immunohistochemistry figure of ccRCC sample in the Human Protein Atlas. **C.** Disease free survival of patients with differential GDF15 expression level in TCGA-KIRC dataset. **D.** Overall survival of patients with differential GDF15 expression level in TCGA-KIRC dataset. *** p < 0.001

We then sought to validate GDF15 downregulation in clinical specimens and explore its prognostic value. qRT-PCR was used to analyze the expression level of GDF15 in eight pairs of ccRCC tissue samples and the corresponding adjacent normal tissue samples (Additional file 5: Table S1). In line with our results of bioinformatic analyses, GDF15 was downregulated in ccRCC tumor samples at both protein (Fig. 2A-B) and mRNA level (Additional file 1: Figure S1A), suggesting a potential anti-tumor role in ccRCC. Furthermore, Kaplan–Meier survival analysis was used to estimate survival outcome of ccRCC, which revealed that patients with overexpressed GDF15 had significantly longer disease-free survival (Fig. 2C, logrank p = 0.016). Similarly, patients with overexpressed GDF15 tended to have longer overall survival than those with lowly expressed GDF15, despite that statistical significance was not met (Fig. 2D, logrank p = 0.1). Collectively, these results verified the decreased expression of GDF15 in ccRCC, which is correlated with disease relapse and unfavorable survival expectancy.

### GDF15 downregulation enhances tumor aggressiveness and contributes to ccRCC progression

**Fig. 3:**
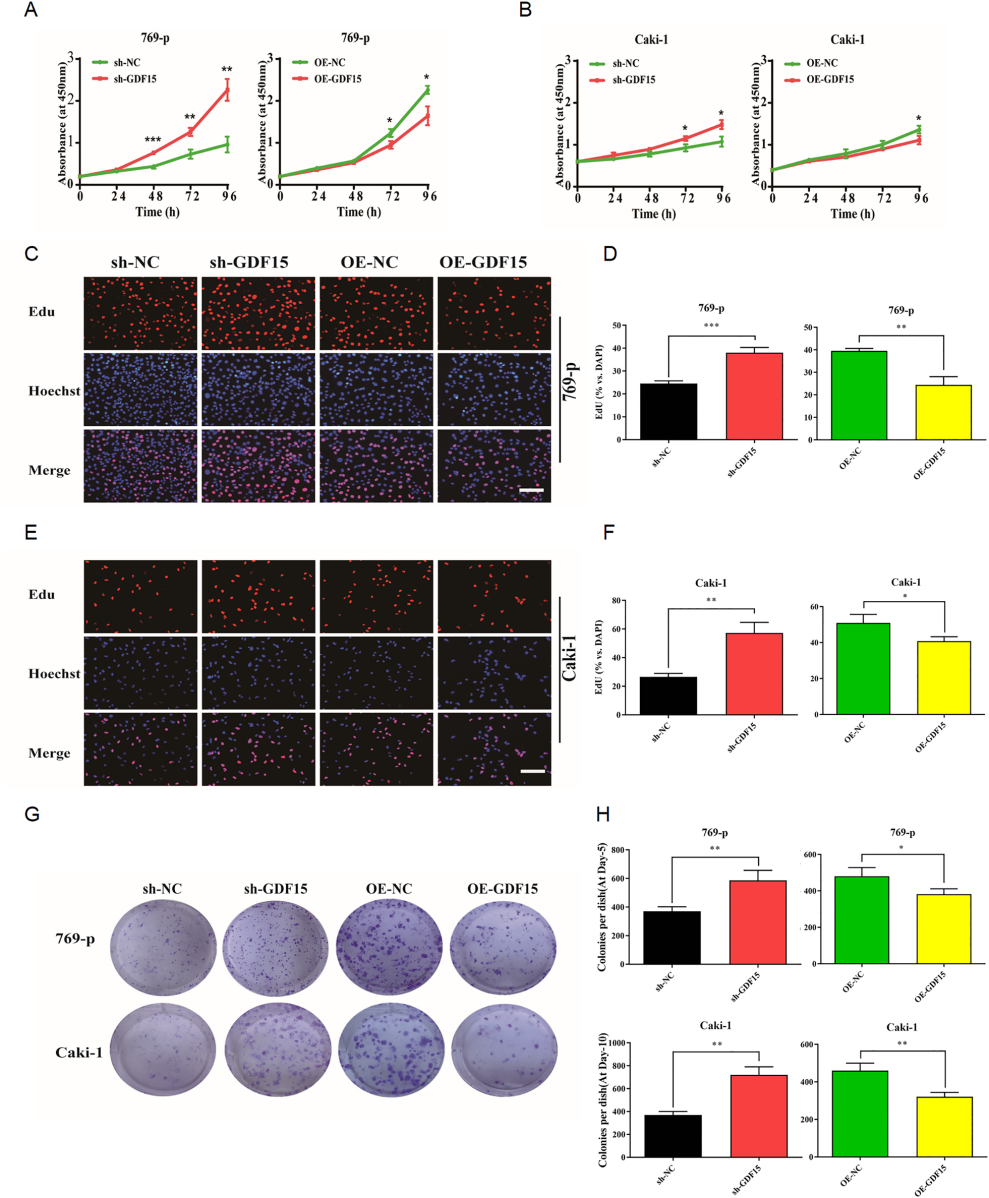
Downregulation of GDF15 promotes the growth and proliferation of ccRCC. **A**. Line plots showing the results of CCK-8 assay performed in 769-p cells. **B**. Line plots showing the results of CCK-8 assay performed in Caki-1 cells. **C**. Images showing the results of EdU assays performed in 769-p cells. **D**. Bar plots showing the statistical analysis of the results of invasion assays performed in 769-p cells. **E**. Images showing the results of Edu assays performed in Caki-1 cells. **F**. Bar plots showing statistical analysis of the results of EdU assays performed in Caki-1 cells. **G**. Images showing the images of colony formation assay. **H**. Bar plots showing the statistical analysis of the results of colony formation assays performed in 769-p and Caki-1 cells. * p < 0.05, ** p < 0.01, *** p < 0.001

We then sought to investigate cellular phenotypes associated with altered GDF15 expression levels in ccRCC. 769-p and Caki-1 cell lines with stable overexpression or knockdown of GDF15 were established (Additional file 1: Figure S1B). Results from Cell Counting Kit-8 (CCK-8) assay demonstrated that 769-p (Fig. 3A) and Caki-1 (Fig. 3B) cells with decreased GDF15 expression had enhanced viability compared to cells in the control knockdown group, while ectopic expression of GDF15 hindered viability of these tumor cells. Moreover, we observed that knockdown of GDF15 in both cell lines significantly increased EdU-positive ratio while forced expression of GDF15 led to decreased EdU-positive ratio (Fig. 3C-F). Since cell proliferation is generally associated with phase changes in the cell cycle, we further investigated the cell cycle of GDF15-modified ccRCC cells using flow cytometry. In our experiments, we found that knockdown of GDF15 resulted in increased proportion of S phase cells and decreased proportion of G0/G1 phase cells (Additional file 2: Figure S2). Conversely, GDF15 overexpression significantly increased the proportion of G0/G1 cells and decreased the proportion of S phase cells (Additional file 2: Figure S2). These results suggested that GDF15 inhibits ccRCC cell proliferation by delaying the transition from G0/G1 to S phase of the cell cycle.

**Fig. 4:**
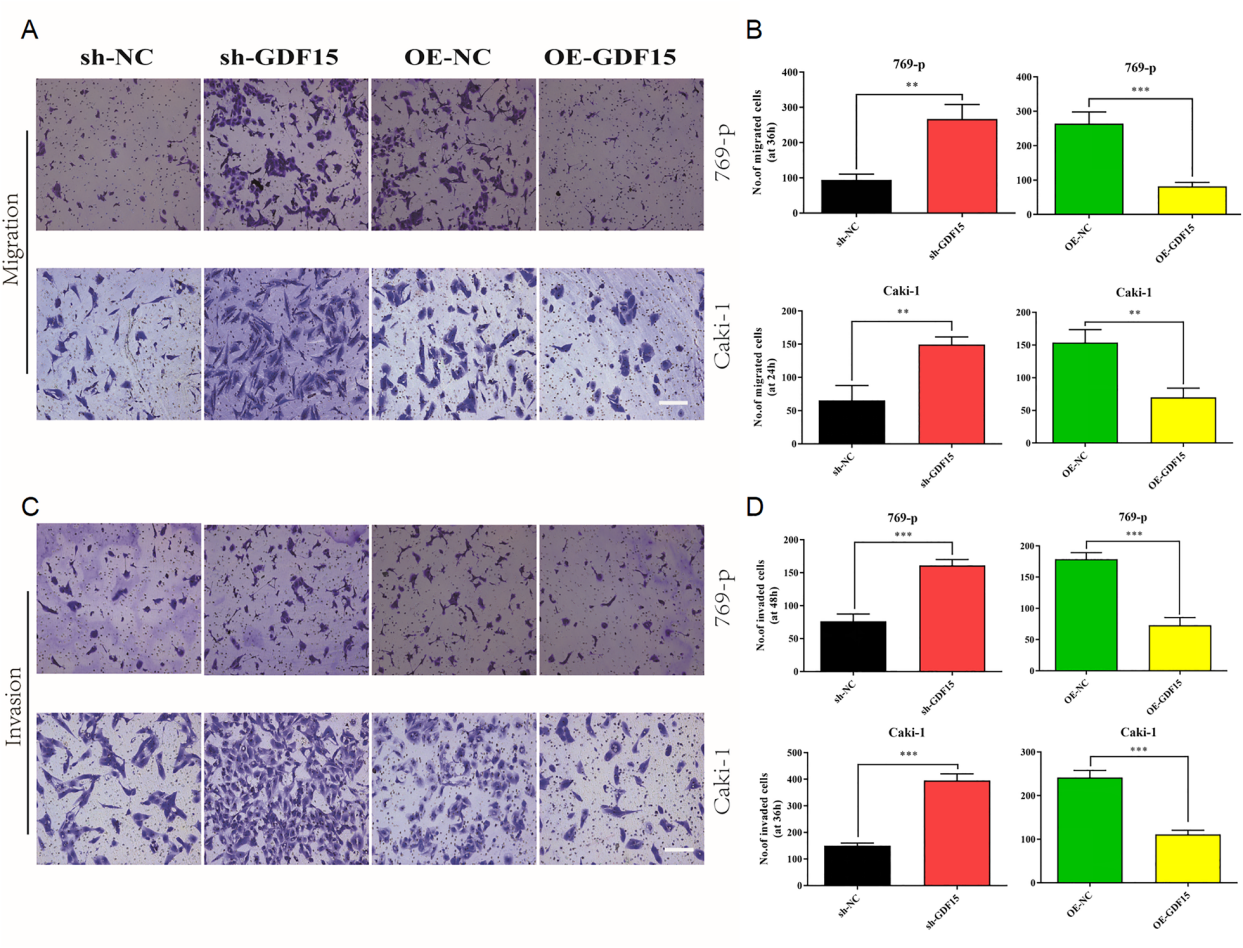
Downregulation of GDF15 promotes the aggressiveness of ccRCC. **A**. Images showing the results of migration assays performed in 769-p and Caki-1 cells. **B.** Bar plots showing the statistical results of migration assays performed in 769-p and Caki-1 cells. **C.** Images showing the results of invasion assays performed in 769-p and Caki-1 cells. **D.** Bar plots showing the statistical results of invasion assays performed in 769-p and Caki-1 cells. * p < 0.05, ** p < 0.01, *** p < 0.001

In order to further assess the role of GDF15 in regulating ccRCC aggressiveness, we conducted colony formation assay (Fig. 3G-H), wound-healing assay (Additional file 3: Figure S3), and Transwell assays (Fig. 4A-D). GDF15 knockdown in both cells promoted colony formation and accelerated cell migration and invasion significantly. In summary, GDF15 plays an inhibitory role in regulating ccRCC aggressiveness.

### GDF15 promotes ferroptosis of ccRCC cells

**Fig. 5:**
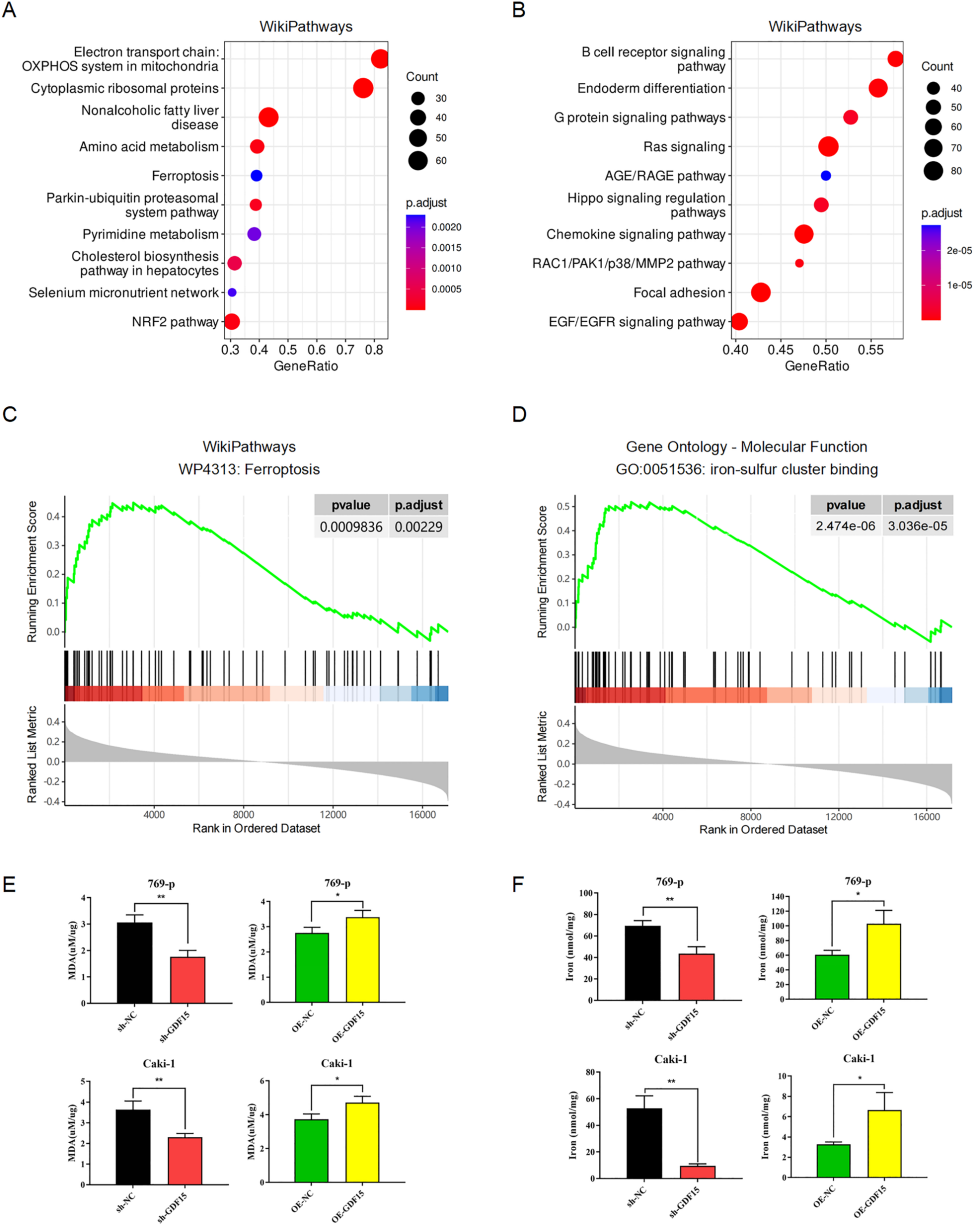
GDF15-related genes are enriched in biological pathways associated with iron metabolism and ferroptosis. **A**, **B**. Dot plots showing the results of significantly enriched pathways by Gene Set Enrichment Analysis (GSEA) that was positively (**A**) or negatively (**B**) correlated with GDF15 expression, where dot size and color represent the number of gene entities included in a given pathway term and the adjusted p value, respectively. C-D. GSEA plots exhibiting selected enriched pathways in WikiPathways (**C**) and Gene Ontology—Molecular Function (**D**) knowledgebases. **E** Bar plots showing the results of MDA assay performed in 769-p and Caki-1 cells. **F** Bar plots showing the results of iron assay performed in 769-p and Caki-1 cells. * p < 0.05, ** p < 0.01

To characterize molecular functions underlying phenotype shifts associated with altered GDF15 expression, genes are ranked and ordered based on their correlation with GDF15 in TCGA-KIRC dataset and were subjected to Gene Set Enrichment Analysis (GSEA) to infer significantly enriched signaling pathways in an unbiased fashion. Pathways in WikiPathways knowledgebase that were significantly correlated with the GDF15 high expression included those associated with mitochondrial functions, metabolic reprogramming, and oxidative phosphorylation (Fig. 5A). Cancer cells tend to reprogram glucose metabolism and limit energy fueling largely to glycolysis, a hallmark that has been famously termed ‘aerobic glycolysis’ by Otto Warburg [18]. Therefore, a metabolic switch towards mitochondrial oxidative phosphorylation could partially explain the correlation between GDF15 expression and a less aggressive phenotype. On the contrary, GDF15 low expression was associated with a number of pathways known to promote cancer growth and progression (Fig. 5B), such as G protein signaling pathways, Ras signaling, Hippo signaling regulation pathways, chemokine signaling pathway, RAC1/PAK1/p38/MMP2 pathway, Focal adhesion, EGF/EGFR signaling pathway, etc. We also performed GSEA with reference to Reactome knowledgebase and found similar results (Additional file 4: Figure S4A, B). These results were in line with our observations that GDF15 knockdown not only promoted tumor proliferation but also contributed to tumor invasion and metastasis.

**Fig. 6:**
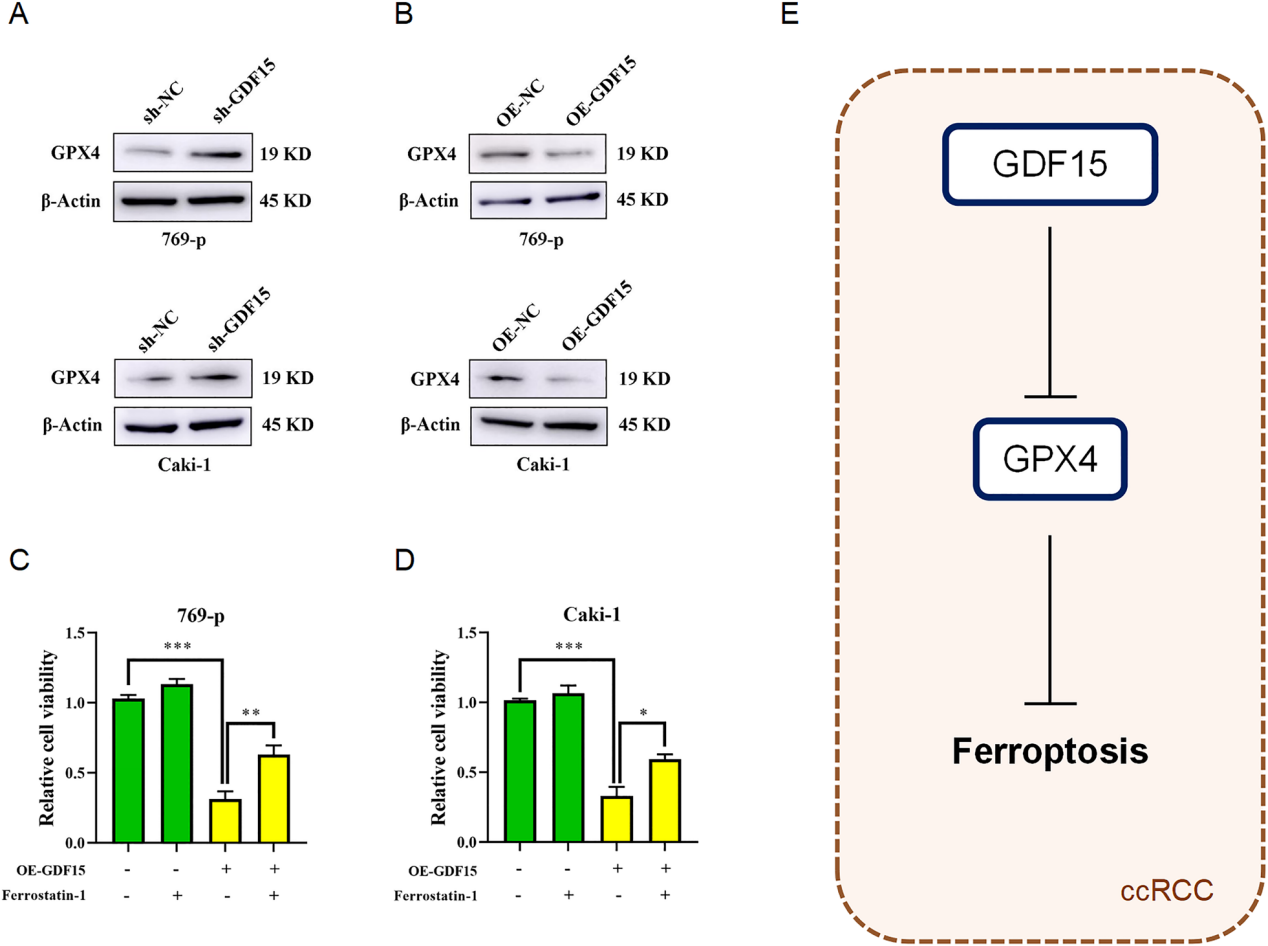
GDF15 is co-expressed with GPX4 and promotes ferroptosis. **A**. Western blotting images showing the effect of GDF15 knockdown on GPX4 expression in 769-p and Caki-1 cell lines. **B**. Western blotting images showing the effect of GDF15 forced over-expression on GPX4 expression in 769-p and Caki-1 cell lines. **C**, **D**. Line plots showing the results of cell proliferation in GDF15-overexpressed 769-p (**C**) and Caki-1 cells (**D**). **E** Schematic diagram of the molecular mechanisms linking GDF15 to ferroptosis. * p < 0.05, ** p < 0.01, *** p < 0.001

Interestingly, we noticed that Ferroptosis (WP4313), which has recently attracted much attention in the cancer research community, was significantly enriched among the pathways that were positively correlated with GDF15 expression (Fig. 5A, C). GSEA performed using other knowledgebases also discovered significantly enriched iron metabolism related pathways that were associated with increased GDF15 expression, including iron-sulfur cluster binding (GO:0051536) in Gene Ontology (Fig. 5D) and Iron update and transport, Mitochondrial ion-sulfur cluster biogenesis, and Transferrin endocytosis and recycling in Reactome (Additional file 4: Figure S4C). We therefore proposed that ferroptosis might play a significant role in GDF15-induced inhibition of ccRCC progression. MDA and iron have been proven to be vital metabolic indicators for ferroptosis regulation. We measured intracellular levels of MDA and iron in GDF15-perturbed ccRCC cells and found that GDF15 overexpression significantly increased MDA and iron levels, whereas knockdown of GDF15 resulted in decreased MDA and iron levels (Fig. 5E-F). We also checked the expression pattern of GDF15 and known regulators of ferroptosis. By performing Western blotting, we experimentally validated the negative correlation between GDF15 and GPX4 in multiple cell lines (Fig. 6A-B). Futhermore, we found that GDF15 overexpression resulted in increased cell death of 769-p and Caki-1 cells, while treatment with ferrostatin-1, an inhibitor of ferroptosis, significantly attenuated cell death, indicating that GDF15 attenuated cell growth by promoting ferroptosis (Fig. 6C-D). Taken together, these findings suggest that GDF15 promotes ferroptosis in ccRCC cells, which could be mediated by GPX4 (Fig. 6E).

## Discussion

Ferroptosis is an iron-dependent form of controlled cell death characterized by an excess of lipid peroxides [19]. It is characterized by cell death which is primarily mediated by iron and lipid peroxidation [20]. Ferroptosis plays a role in a number of pathological diseases, including ischemia reperfusion injury, neurodegeneration, and cancer [21, 22]. In the field of oncology, ferroptosis reportedly regulates the development, metastasis, and invasion in several cancer types [23]. Recently, the induction of ferroptosis has been considered as a potential therapeutic strategy for treating cancer. For example, the activation of ferroptosis greatly increased the sensitivity of gemcitabine, suggesting potential therapeutic strategies for the treatment of pancreatic cancer [24]. Liu, et al. [25] reported that SIRT3 drives AKT-dependent ferroptosis and inhibited epithelial-mesenchymal transition in gallbladder cancer cells. Talaroconvolutin A, a novel inducer of ferroptosis, effectively inhibited the growth of xenogeneic colorectal cancer cells by downregulating SLC7A11 and upregulating ALOXE3 in vivo experiments [16]. Ferroptosis has been reported not infrequently in RCC. Lu, et al*.*[26] found that Lipro, a ferroptosis-specific inhibitor, restores the migratory and invasive ability of 786-O cells when GDF15 was overexpressed. In this study, we utilized TCGA-KIRC dataset to perform pathway enrichment analysis, in which pathways related to iron metabolism and ferroptosis stood out. We then measured intracellular MDA and iron levels in stable transfected cells and found that GDF15 overexpression significantly increased MDA and iron levels, which experimentally supported the activation of ferroptosis following GDF15 overexpression.

Ferroptosis is closely related to numerous biological processes and intrinsically regulated by complex signaling pathways. GPX4 and SLC7A11 are considered to be major players regulating ferroptosis. GPX4, an inhibitor of lipid peroxidation, can provide reducing equivalents to eliminate oxidation by regulating the cellular antioxidant system downstream of the signal [25]. A recent study [26] reported that GPX4 was closely associated with ccRCC and conferred ferroptosis sensitivity to cancer cells. In our study, we found that GDF15 was positively correlated with GPX4, but not SLC7A11, ATF1, or AKT1. As a result, we proposed that GDF15 positively regulates ferroptosis, which could be mediated by GPX4.

As a member of the TGF-β superfamily, GDF15 is involved in various pathophysiological processes. Research has demonstrated that GDF15 plays an important role in regulating the properties of a variety of cancer cells and has a considerable clinical significance for patients with cancer. Studies on the role of GDF15 in tumors are not uncommon, but the function of GDF15 in different cancer types is controversial. Cekanova, et al. [27] discovered that GDF15 inhibited urethane-induced tumor formation via regulating the p38/MAPK signal pathway. However, Li, et al. [28] found that knockdown of GDF15 in cervical cancer inhibits cell progression via the TGF-β/Smad2/3/Snail1 pathway. Our results clearly showed that, while GDF15 is usually upregulated in most cancers, it is downregulated in kidney cancers, especially in ccRCC. These results were consistent with the predictions of Caceres et al. [29], who proposed that GDF15 is hypermethylated in ccRCC cells relative to normal renal cells. In our study, GDF15 overexpression suppressed the migratory and invasive activity of RCC cells. By contrast, GDF15 knockdown increased the aggressiveness of 769-p and Caki-1 cell. In summary, our results indicated that GDF15 is an important tumor suppressor gene in ccRCC.

There were some limitations in our study. First, our results were derived from clinical sample analysis and in vitro experiments. We did not perform animal studies to validate these results in vivo. Second, we were unable to experimentally identify a single most important mediator linking GDF15 to ferroptosis. However, we are dubious about whether such a mediator molecule even exists. Indeed, components in the signaling transduction pathways are often intricately networked and intertwined, and attempts to simplify a complex biological process to a few key driver molecules would most likely lead to misinterpretation of the nature of the compound. Third, we did not explore the in-depth mechanistic details on GDF15 regulating ferroptosis in the current study, which focused on the prioritization of GDF15 in ccRCC and the discovery of its role in regulating ferroptosis. We anticipate to further address these questions in future studies.

## Conclusion

Taken together, our study showed that GDF15 could inhibit the proliferation, migration, invasion and induce ferroptosis of ccRCC cells, potentially by upregulating GPX4, and that downregulation of GDF15 could led to unfavorable patient survival. Our findings may provide a novel therapeutic target for ccRCC. This study also exemplified a useful framework for analyzing the molecular functions of genes of interest in the context of a particular disease or tumor type.

## Methods

### Human kidney cancer tissue specimen acquisition

Primary ccRCC tissues and adjacent normal tissue specimens were collected for quantitative real-time PCR (qRT-PCR) from the Affiliated Zhangjiagang Hospital of Soochow University (Suzhou, China) from 2016 to 2021. The cancerous tissue was pathologically confirmed as clear cell carcinoma and staged according to the American Joint Committee on Cancer classification (AJCC) in its eighth edition. Prior to clinical information and specimen collection, each patient provided informed consent.

### Cell culture and transfection

The human ccRCC cell lines 769-p and Caki-1 were obtained from the Cell Bank of Shanghai Institute of Biochemistry and Cell Biology (Shanghai, China). 769-p cells were cultivated in RPMI-1640 medium (Gibco, Carlsbad, USA) and Caki-1 cells were cultured in McCoy 5A medium (Gibco). Both the recommended medium included 10% fetal bovine serum (FBS, Vivacell, Shanghai, China) and 1% penicillin/streptomycin (Gibco), and both cell lines were cultured at 37 °C in an incubator with 5% CO2. Genechem Co., Ltd (Shanghai, China) constructed and authenticated the shRNA (small hairpin RNA) and overexpression lentiviruses for GDF15, and the correspondingly empty vectors (sh-NC/OE-NC) served as controls. GDF15 shRNA lentiviral vectors were derived from GV248 plasmids, with the target sequence designed as follows:GAGUUGCACUCCGAAGACU. Negative control shRNA lentiviral vectors were Ubi-MCS-3FLAG-SV40-EGFP-IRES-Puro which were derived from GV358 plasmids. 769-p and Caki-1 cells were seeded at a density of 1*10^5^ cells per well in 6-well plates (Costar, USA) and cultivated for 24 h. Afterward, the cells were transfected with constructed lentivirus for 16 h, during which the culture medium was changed depending on the cell activity. Stable transfected cells were screened with puromycin after 72 h of infection.

### qRT-PCR

TRIzol reagent (Invitrogen) was used to extract total RNA from tissues and cells. Through reverse transcription, cDNA was obtained using the PrimeScript RT Reagent Kit (ABI, USA). qRT-PCR was performed using PowerUp^™^ SYBR Green Master Mix (ABI, USA). The expression of GDF15 was quantified using the 2^^−ΔΔCt^ method and GAPDH was used as an endogenous control. The primer pairs were used for qRT-PCR as follow: GDF15 forward primer, GCTACGAGGACCTGCTAACC, reverse primer, GCACTTCTGGCGTGAGTATC; GAPDH forward primer, CAGGAGGCATTGCTGATGAT, reverse primer, GAAGGCTGGGGCTCATTT.

### Western blotting

RIPA lysis buffer (Beyotime) containing phosphatase inhibitor cocktail A (PMSF, Beyotime) was used to extract total protein. BCA assays kit (Beyotime) was used to quantify the proteins before loading them into 4–20% FuturePAGEMT protein preformed gels (ACE, Nanjing, China) for western blot. The target proteins were transferred to polyvinylidene fluoride membrane. After blocking with 5% BSA, the membrane was incubated using primary antibodies overnight at 4℃, which was followed by secondary antibodies incubation for 2 h at room temperature. The ChemiDoc™ Touch Imaging System (BIO-RAD, USA) was used to analyze the blots for chemiluminescence using Immobilon western chemilum HRP Substrate (Millipore, Massachusetts, USA). Primary antibodies used in this study were GDF15 (abcam, ab206414, 1:1000), GPX4 (abcam, ab125066, 1:1000), and β-actin (proteintech, 81,115–1-RR, 1:2000).

### Cell viability and wound-healing assay

96-well plates (Costar) were seeded with 5000 cells per well and incubated for 24, 48, 72, and 96 h. Each well was filled with 110-μl of new media containing 10-μl of Cell Counting Kit-8 (CCK-8) reagent (Dojindo, Kumamoto, Japan) and incubated at 37 °C for 2 h. Using the CYTATION 5 Imaging Reader (BioTek, USA), the absorbance of each well was measured at 450 nm. We conducted all experiments in triplicate. In the wound healing assay, 5*10^5^ cells were seeded into a 6-well plate. The next day, the surface of the cells was wounded with a 10-μl sterile gun tip (Kirgen, USA) in a vertical monolayer. After wounding, the non-adherent cells were washed away with phosphate buffered saline (Meilunbio, Dalian, China) and replaced with fresh serum-free medium. The degree of wound healing was assessed using a light microscope.

### Cell migration and invasion assay

A serum-free cell suspension containing 4 × 10^4^ 769-p cells and 6 × 10^4^ Caki-1 cells was seeded into the upper chamber of Transwell (Falcon, USA), while complete medium containing 10% FBS was seeded into the lower chamber. After incubation in an incubator at 37 °C for 24 h, cells were fixed with 4% paraformaldehyde (Solarbio, Peking, China) for 30 min and stained with crystal violet staining solution (Beyotime Biotech) for 10 min. Briefly, for the invasion experiments, diluted matrigel matrix gel (Corning) was added to the upper chamber of the Transwell, and then the same experimental steps described above were used.

### Sulforhodamine B assay

Sulforhodamine B assay (abcam, USA) was performed according to the manufacturer’s instructions. In brief, cells were seeded into 96-well plates (1*10^3^ cells/well) and incubated overnight at 37 ℃. After treatment with ferrostatin-1 or DMSO, cells were fixed and stained. CYTATION 5 Imaging Reader was used to assess the cell viability at 560 nm.

### Colony formation and 5-ethynyl-20-deoxyuridine (EdU) assay

For colony formation assay, 1*10^3^ treated cells were seeded into 6-well plates. Following a 10-day incubation period, the plates were washed twice with PBS, fixed in 4% paraformaldehyde for 30 min, and stained with crystal violet staining solution for 10 min. Cell proliferation ability was detected by EdU assay kit (Ribobio, Yangzhou, China). Cells were seeded into 24-well plates at a density of 10^5^ cells per well. After 16 h, cells were cultured with 50-μM EdU buffer for 2 h in a constant-temperature incubator before being fixed with 4% paraformaldehyde for 30 min. Excess aldehyde groups were then neutralized with 2 mg/ml glycine and cell membranes were permeabilized using 0.5% Triton X-100 for 10 min. Apollo staining reaction solution from EdU kit was added into each well, and then the nuclei were stained with Hoechst. Finally, the results were observed by fluorescence microscopy.

### Cell cycle analysis

Cells were collected and fixed with cooled 75% ethanol at −20℃ for at least one night. After discarding the ethanol, cells were washed twice with PBS and resuspended at room temperature for 30 min with 250 μl DNA staining solution (Multiscience, China). Flow cytometry (FACS LSRII, BD Bioscience, USA) was used to analyse the cell cycle. Propidium iodide was used for DNA staining.

### Evaluation of glutathione (GSH), malondialdehyde (MDA) and iron levels

Concentrations of GSH, MDA, and iron were measured using the GSH Kit (Beyotime), Lipid Peroxidation (MDA) Assay Kit (Beyotime), and Iron Assay Kit (Abcam), respectively, according to the manufacturer's instructions.

## Statistical analysis

Each experiment was replicated at least three times. The continuous data were presented as mean ± standard deviation (SD). Unless otherwise specified, the statistical significance between two groups of continuous data was examined using two-sided, independent samples t-tests or Wilcoxon tests. The statistical significance between different survival curves was examined through logrank test. For DFS analysis of TCGA-KIRC, cox proportional hazards regression model was used to calculate hazard ratio (HR). The significance threshold alpha was set as 0.05.

### Supplementary Information

**Additional file 1: Figure S1.** Expression of GDF15 in tissue samples and cancer cell lines. **A** Bar plots showing GDF15 mRNA expression in different types of tissue samples. T, tumor tissue samples. N, adjacent normal tissue samples. B. Western blotting images showing efficacy of GDF15 shRNA knockdown and GDF15 forced overexpression in 769-p and Caki-1 cancer cell lines.

**Additional file 2: Figure S2.** Cell cycle analysis. **A** Representative density plots of cell cycle analysis using flow cytometry. **B**. Bar plots showing the results of cell cycle analysis in perturbed 769-p and Caki-1 cells.

**Additional file 3: Figure S3.** Wound healing assay. Representative images of wound healing assay performed in perturbed 769-p and Caki-1 cells.

**Additional file 4: Figure S4.** Reactome pathways enriched by GDF15-related genes. Dot and tree plots showing the results of significantly enriched pathways identified in the ReactomeDB by Gene Set Enrichment Analysis (GSEA) that was positively (A) or negatively (B) correlated with GDF15 expression, where shaded colors represent different clusters of pathways which share semantic similarity and dot size and color represent the number of gene entities included in a given pathway term and the adjusted p value, respectively. C. GSEA plots exhibiting selected enriched pathways in ReactomeDB.

**Additional file 5: Table S1.** Patient sample clinical information.

## Data Availability

The TCGA PANCAN dataset is available at UCSC Xena Data Hub (https://xenabrowser.net/). Other data generated during this study were included in this article.
