# Supplementary figures and images for: Downregulation of GDF15 suppresses ferroptosis and predicts unfavorable prognosis in clear cell renal cell carcinoma
Source: Cell Div. 2023 Dec 11;18:21. doi: 10.1186/s13008-023-00103-9 (PMC10712134; doi:10.1186/s13008-023-00103-9)

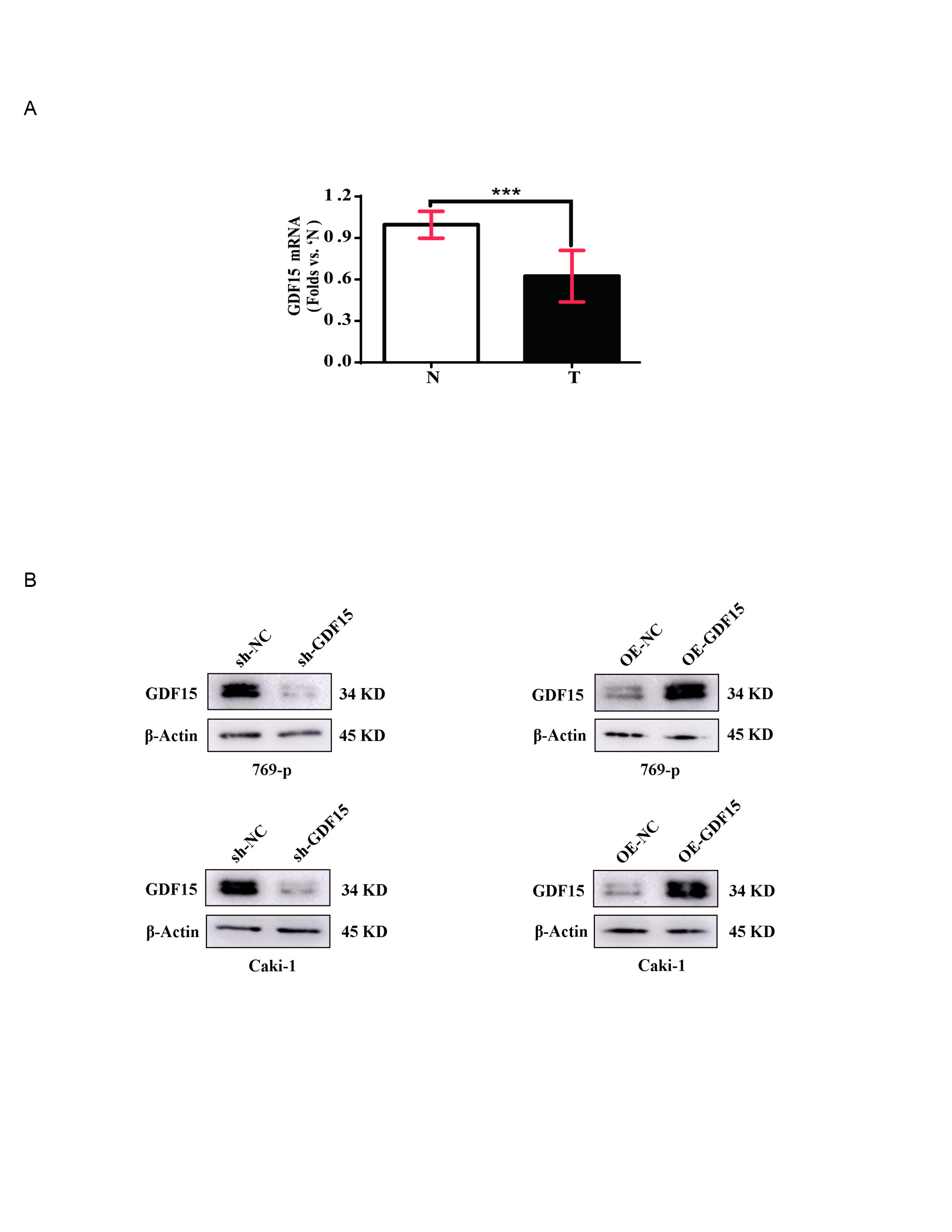

Supplement: Supplementary file 1 — Additional file 1: Figure S1. Expression of GDF15 in tissue samples and cancer cell lines. A Bar plots showing GDF15 mRNA expression in different types of tissue samples. T, tumor tissue samples. N, adjacent normal tissue samples. B. Western blotting images showing efficacy of GDF15 shRNA knockdown and GDF15 forced overexpression in 769-p and Caki-1 cancer cell lines. [file 13008_2023_103_MOESM1_ESM.tif]

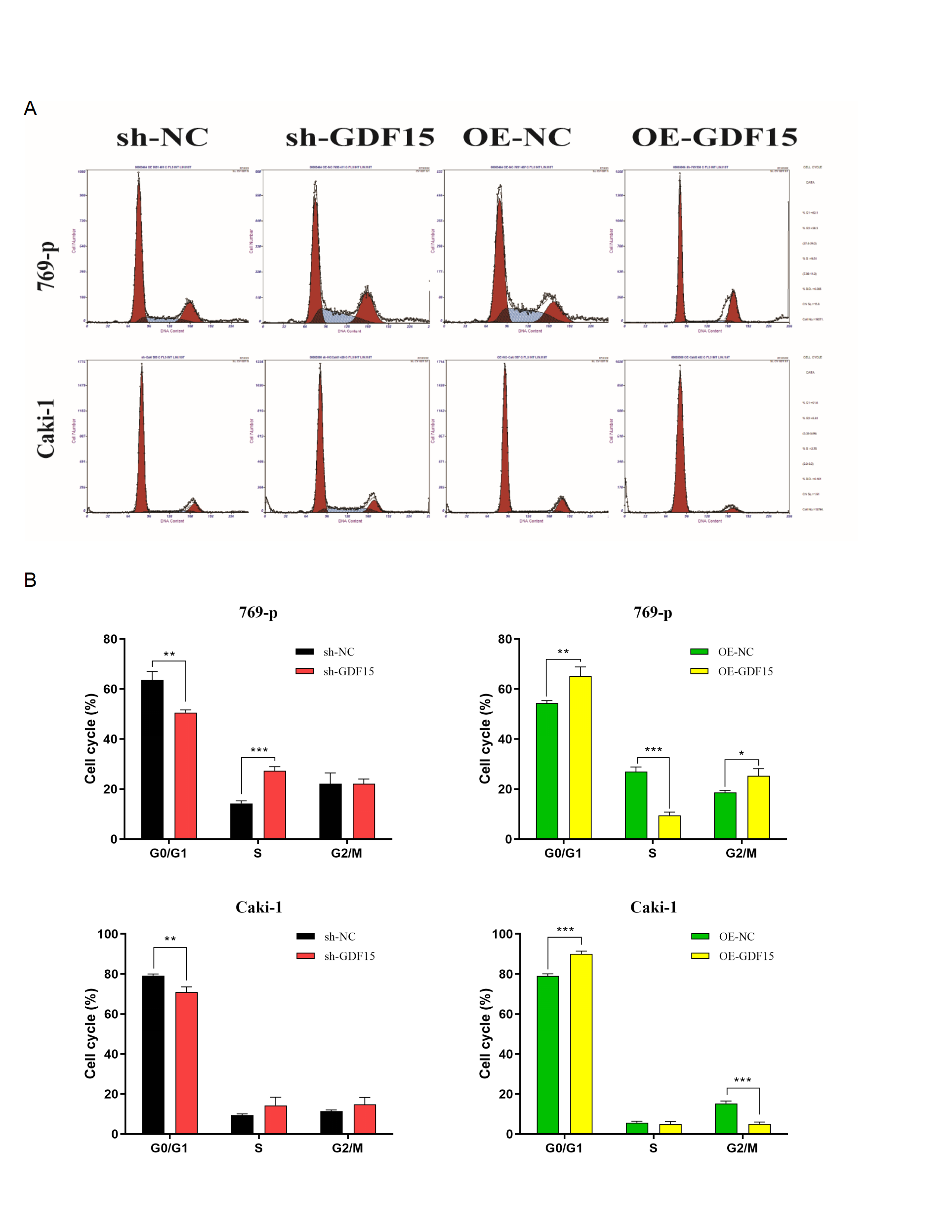

Supplement: Supplementary file 2 — Additional file 2: Figure S2. Cell cycle analysis. A Representative density plots of cell cycle analysis using flow cytometry. B. Bar plots showing the results of cell cycle analysis in perturbed 769-p and Caki-1 cells. [file 13008_2023_103_MOESM2_ESM.tif]

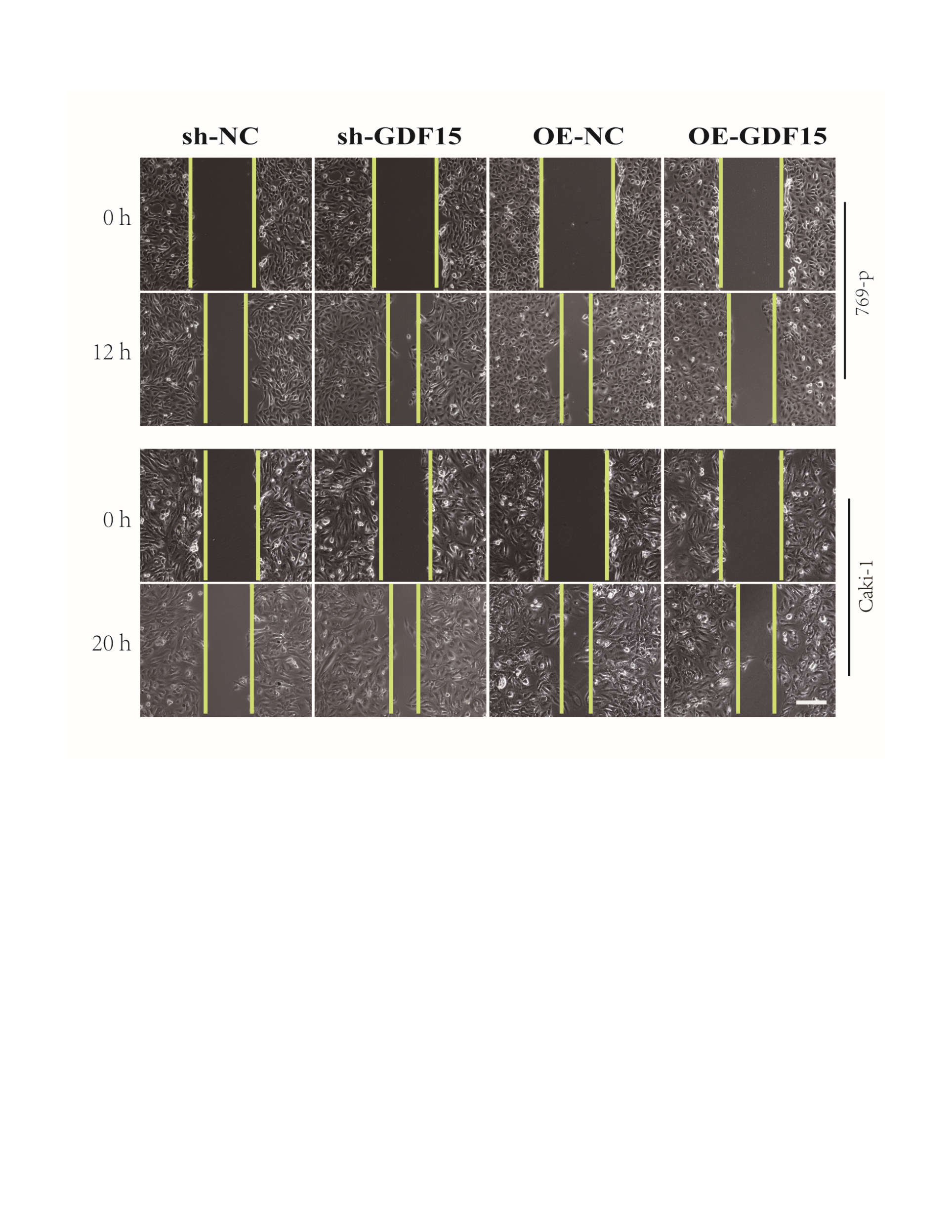

Supplement: Supplementary file 3 — Additional file 3: Figure S3. Wound healing assay. Representative images of wound healing assay performed in perturbed 769-p and Caki-1 cells. [file 13008_2023_103_MOESM3_ESM.tif]

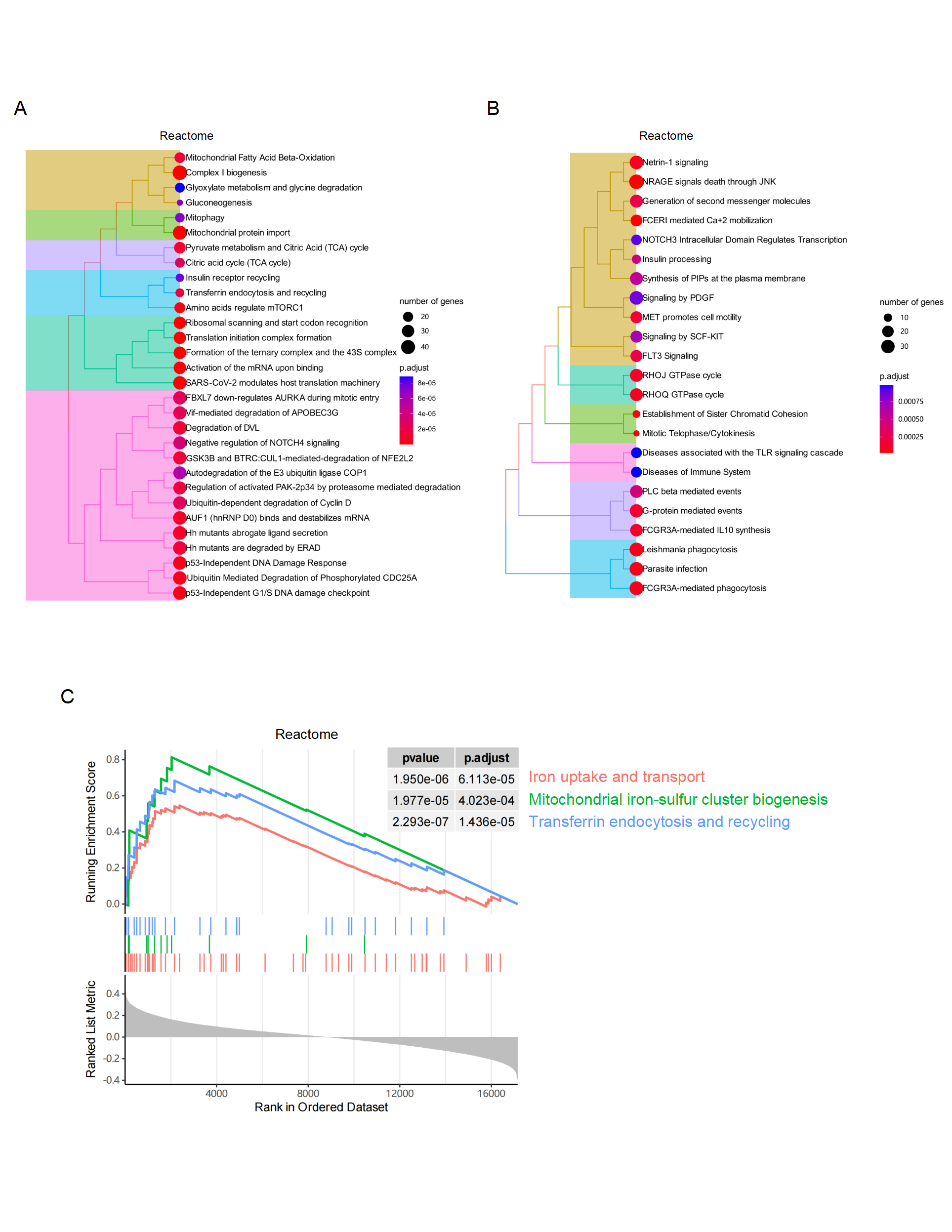

Supplement: Supplementary file 4 — Additional file 4: Figure S4. Reactome pathways enriched by GDF15-related genes. Dot and tree plots showing the results of significantly enriched pathways identified in the ReactomeDB by Gene Set Enrichment Analysis (GSEA) that was positively (A) or negatively (B) correlated with GDF15 expression, where shaded colors represent different clusters of pathways which share semantic similarity and dot size and color represent the number of gene entities included in a given pathway term and the adjusted p value, respectively. C. GSEA plots exhibiting selected enriched pathways in ReactomeDB. [file 13008_2023_103_MOESM4_ESM.tif]
