# Supplementary material for: Downregulation of GDF15 suppresses ferroptosis and predicts unfavorable prognosis in clear cell renal cell carcinoma
Source: Cell Div. 2023 Dec 11;18:21. doi: 10.1186/s13008-023-00103-9 (PMC10712134; doi:10.1186/s13008-023-00103-9)
Supplement: Supplementary file 5 — Additional file 5: Table S1. Patient sample clinical information. [file 13008_2023_103_MOESM5_ESM.docx]

**Additional Table 1. Patient sample clinical information.**

| Participant ID | Sex | Age | Preoperative serum creatine level (μmol/L) | Clinical staging | Pathological diagnosis | Fuhrman Nuclear Grade |
| --- | --- | --- | --- | --- | --- | --- |
| Z1 | Female | 53 | 86 | T2aN0M0 | ccRCC | Ⅱ |
| Z2 | Male | 69 | 60 | T1aN0M0 | ccRCC | Ⅱ |
| Z3 | Female | 45 | 110 | T1aN0M0 | ccRCC | Ⅱ |
| Z4 | Male | 50 | 74 | T3aN0M0 | ccRCC | Ⅱ - Ⅲ |
| Z5 | Male | 43 | 66 | T1aN0M0 | ccRCC | Ⅱ |
| Z6 | Female | 55 | 67 | T3aN0M0 | ccRCC | Ⅲ |
| Z7 | Male | 57 | 59 | T1aN0M0 | ccRCC | Ⅱ |
| Z8 | Female | 66 | 62 | T1aN0M0 | ccRCC | Ⅱ |

ccRCC, clear cell renal cell carcinoma.
